# A Novel Feed Additive Derived from Mushroom-Based Phytonutrients: Effects on Rumen Antioxidant Capacity, Fermentation, and Methane Production

**DOI:** 10.3390/vetsci13060554

**Published:** 2026-06-03

**Authors:** Maharach Matra, Eakapol Wangkahart, Burarat Phesatcha, Sukruthai Sommai, Metha Wanapat

**Affiliations:** 1 Applied Animal and Aquatic Sciences Research Unit, Division of Animal Science, Department of Agricultural Technology, Faculty of Technology, Mahasarakham University, Maha Sarakham 44150, Thailand; maharach.m@msu.ac.th; 2 Laboratory of Fish Immunology and Nutrigenomics, Applied Animal and Aquatic Sciences Research Unit, Division of Fisheries, Faculty of Technology, Mahasarakham University, Maha Sarakham 44150, Thailand; 3 Department of Applied Biology, Faculty of Sciences and Liberal Arts, Rajamangala University of Technology Isan, Nakhon Ratchasima 30000, Thailand; burarat.ph@rmuti.ac.th; 4 Department of Animal Science, Faculty of Agricultural Technology, Buriram Rajabhat University, Buriram 31000, Thailand; sukruthai.sm@bru.ac.th; 5 Tropical Feed Resources Research and Development Center, Department of Animal Science, Faculty of Agriculture, Khon Kaen University, Khon Kaen 40002, Thailand

**Keywords:** phytogenic feed additive, fungi, eco-friendly natural resources, antioxidant activity, ruminants

## Abstract

Mushrooms are considered a significant source of antioxidants, antimicrobials, and phytonutrients. Consequently, they can serve as a phytogenic feed additive to prevent oxidative stress, while improving rumen fermentation efficiency. The aim of the present investigation was to evaluate the effects of bolete mushroom extract (BME) supplementation on in vitro digestive enzymes, nutrient degradability, antioxidant capacity, rumen fermentation, and methane production. In a completely randomized design, six treatment levels of BME were implemented. The total dry matter (DM) substrate of BME was supplemented at 0%, 1%, 2%, 3%, 4%, and 5%. The results demonstrated that BME increased the activity of digestive enzymes, particularly amylase activity and in vitro dry matter degradability. Importantly, at 4% of the total DM substrate, BME significantly increased levels of total antioxidant and catalase activity, while malondialdehyde activity was reduced. BME addition contributed to an increase in propionate and total volatile fatty acid production, while methane production was decreased. As a result, BME could be effective as a sustainable feed additive comprising phytonutrients to enhance rumen fermentation and reduce methane emissions in ruminants.

## Simple Summary

Oxidative stress has been researched for its detrimental impact on the immune and reproductive systems, encompassing key aspects of animal performance. Mushrooms are considered a significant source of antioxidants and phytonutrients. As a result, they can serve as a phytogenic feed additive to prevent oxidative stress, while improving rumen fermentation efficiency. In this study, bolete mushroom extract supplementation at 4% of the total dry matter substrate can simultaneously enhance rumen enzyme activity, antioxidant capacity, fermentation efficiency, and decreased methane production. This in vitro study presents a sustainable and eco-friendly approach to feed additives, which requires further validation through in vivo research.

## 1. Introduction

Several stressors contribute to economic losses resulting from diminished productive performance in ruminants, particularly dairy cows. Research indicates that dietary, technical, environmental, and internal stressors result in the excessive generation of free radicals and oxidative stress [1]. Oxidative stress, defined as an imbalance between free radical generation and detoxification, is widely recognized for its adverse effects on immunological and reproductive systems, including critical aspects of animal growth and health [2]. Peroxidation occurs due to the essential process of cellular oxidative metabolism, which generates reactive oxygen species and free radicals that cause oxidative damage [3]. Under typical conditions, cells are safeguarded by antioxidants and intracellular enzymes, including catalase (CAT) and glutathione peroxidase (GPx), which neutralize peroxides to avert the generation of more reactive compounds through reactions with metal catalysts [4]. Currently, feed additives possessing phytonutrient and antioxidant properties are frequently utilized to mitigate oxidative stress and modulate nutrient degradability, rumen fermentation characteristics, and methane production [5,6].

Mushrooms have historically been employed in Asia for medical purposes and as functional foods [7]. Importantly, they are increasingly regarded as a valuable source of antioxidants, anti-inflammatory agents, and phytonutrients (PTNs) [8]. Bolete (*Thaeogyroporus porentosus*) is an important mushroom that has been studied for its phytonutrients, especially phenolics and flavonoids, and is extensively available in Thailand [9]. Phytonutrients may facilitate the shift of hydrogen molecules from the activated hydroxyl group to free radicals, revealing an antioxidant action [10]. These compounds demonstrate diverse biological activity and can enhance rumen fermentation while reducing methane emissions [11]. Kaewnarin et al. [12] reported that bolete mushroom contained total phenolic and flavonoid compounds at 3.3 mg GAE/g DM and 0.7 mg QUE/g DM. A recent investigation by Jia et al. [13] showed that *Agaricus bisporus* mushroom-treated heifers exhibit enhanced antioxidant indexes and feed digestion. Nevertheless, no research has investigated the effects of the PTN-rich bolete mushroom on antioxidant capacity, rumen fermentation efficiency, and methane emissions.

Therefore, the objective of this study was to investigate the impact of the BME supplementation on in vitro digestive enzymes, nutrient degradability, antioxidant capacity, rumen fermentation, and methane production.

## 2. Materials and Methods

### 2.1. Ethics Statement

Protocols were performed in compliance with the guidelines of the Institutional Animal Care and Use Committee of Mahasarakham University (IACUC-MSU), Thailand (Approval no. IACUC-MSU-042-022/2025), and the Institute of Animals for Scientific Purpose Development (IAD), Thailand (Approval no. U1-06878-2560).

### 2.2. Experimental Diets and Chemical Analysis

Experimental feeds consisted of sources of roughage, concentrate, and feed additive are provided in Table 1. The BME preparation used 20 g of mushroom powder, which was extracted using a microwave-assisted extraction method at 450 watts for 30 s for three times in a 1:10 (*w*/*v*) ratio with 80% ethanol. The extraction procedure removes ethanol through evaporation with a rotary evaporator (Buchi Corp., New Castle, DE, USA). To extract the dry powder, the aqueous solution was evaporated after passing it through a Buchner funnel that was lined with Whatman No.1 filter paper. Maltodextrin was added at an amount of 15% to the initial solution. Then, the liquid solution was processed into a powder by freeze-drying (Freeze Dryers, Labconco Corp., Kansas City, MO, USA) utilizing the following conditions: drying air flow (600 L/h), operating speed (10 mL/min), pressure drop (0.75 bar), inlet temperature at 160 °C, and outlet temperature at 90 °C. The liquid was atomized into micron-sized droplets and immediately dried by hot air in the drying chamber, leading to flash evaporation and an accumulation of dried-powder particles. The extracts were placed in glass bottles and stored at −20 °C for subsequent experimental investigation, as demonstrated in the modified procedure by Kanlayavattanakul et al. [14].

Following the protocols of the AOAC [15], concentrate, roughage, and BME were evaluated for ash (method number 942.05), dry matter (method number 967.03), and crude protein (method number 984.13). The proportion of fiber was analyzed utilizing Ankom equipment (Ankom Technology Corp., Macedon, NY, USA) in accordance with the procedure established by Van Soest et al. [16]. The phytonutrients (which include phenolics and flavonoids) present in BME were examined. Total phenolic compounds were quantified using the modified Folin–Ciocalteu technique. In order to establish a calibration curve, standard gallic acid substances were made up in distilled water from a 1 mg/mL stock solution. The absorbance was quantified at 765 nm utilizing a microplate reader (Versamax Microplate Reader, San Jose, CA, USA) [17]. To measure total flavonoid compounds, the standard calibration curve was obtained by utilizing a two-fold serial diminution of standard quercetin in ethanol. The absorbance was quantified at 415 nm using the Versamax Microplate Reader (San Jose, CA, USA) [18].

### 2.3. Donor Animals and Experimental Design

Rumen fluid samples were collected from three Holstein-crossbred dairy cows. The average body weight of the cows was 500 ± 15 kg, and they were two years old. The cows were fed with rice straw ad libitum as a roughage source and a concentrated feed prepared to meet their nutritional requirements. They were fed twice a day at 6:30 a.m. and 4:30 p.m. Before the rumen samples were collected, the cows were fed the diet for 21 days. Prior to the morning feed, samples were administered through the orogastric tube and rapidly transferred to a Thermos bottle for subsequent use.

Six treatment levels of BME were implemented in a completely randomized design. The total DM substrate (from the total basal diet) of the BME was supplemented at 0%, 1%, 2%, 3%, 4%, and 5%, respectively.

### 2.4. Medium Solution Preparation and In Vitro Inoculum

The following are the instructions for combining the components to create approximately 2000 mL of medium solution: add 950 mL of distilled water, 480 mL of buffer solution, 480 mL of macro-mineral solution, 99 mL of reduction solution, 2.44 mL of resazurine, and 0.24 mL of micro-mineral solution, respectively.

In 50 mL bottles, a 60:40 ratio of roughage to concentrate was set with a weight of 0.5 g on a DM basis. Subsequently, the BME samples were weighed at various treatments of total DM substrate. The medium solution was combined with rumen fluid samples at a ratio of 2:1 (mL/mL). Each bottle included 40 mL of artificial saliva solution. Then, bottles with synthetic rubber stoppers were sealed using aluminum caps. All bottles were incubated at 39 °C. A single incubation was run, and all parameters were established with three replicates for each treatment. Samples from the in vitro medium of two sets were collected at 12 and 24 h after incubation for further analysis.

### 2.5. Digestive Enzyme Activity

The activity of amylase was assessed using a modified technique established by Wang et al. [19]. Approximately 1% starch solution and 0.1 M glycine NaOH buffer (pH 8.8) were combined with the in vitro incubation medium sample. The mixture was incubated at 37 °C for 60 min, treated with DNS, heated for 5 min, and diluted with dH_2_O. The absorbance at 550 nm was then measured using an iMark™ Microplate Absorbance Reader (Bio-Rad Laboratories, Inc., Hercules, CA, USA). A modified method of the procedure described by Bezerra et al. [20] was used to measure protease activity. The sample was combined with 1% azocasein and 0.2 M Tris-HCl buffer (pH 7.2). The mixture was then incubated at 37 °C for 60 min, and the reaction was terminated with 20% trichloroacetic acid. Add 1 M NaOH after centrifugation and measure absorbance at 450 nm. A modified protocol described by Iijima et al. [21] was used to measure lipase activity. In this method, the sample was combined with 0.1 M phosphate-buffered saline and 0.1 M p-nitrophenyl palmitate, then incubated at 37 °C for 60 min. The incubation was stopped with 0.1 M Na_2_CO_3_, and absorbance at 405 nm was measured.

### 2.6. Nutrient Degradability

In vitro dry matter degradability (IVDMD) was determined by measuring the residual dry matter after filtering the contents through pre-weighed Gooch crucibles with a porosity of 40 mm. The percentage decrease in weight, following 24 h of drying at 100 °C, was evaluated and recorded as the IVDMD [22].

### 2.7. Antioxidant Capacity and Lipid Peroxidation

The 2,2-diphenyl-1-picrylhydrazyl (DPPH; Sigma-Aldrich Corp., St. Louis, MO, USA) radical scavenging activity was used to evaluate total antioxidants (TAOs), as outlined by Karirat et al. [23]; the iMark™ Microplate Absorbance Reader (Bio-Rad Laboratories, Inc., Hercules, CA, USA) was implemented to measure the optical density (OD) at 520 nm. The measurement of catalase (CAT) was conducted in accordance with the method outlined by Weydert and Cullen [24]; this involved the combination of 10 µL of supernatant with H_2_O_2_ and subsequent measurement at 240 nm. The activity of glutathione peroxidase (GPx) was assessed in alignment with the methodology outlined by Fontagne-Dicharry et al. [25]; 40 μL of freshly prepared phosphate-buffered saline (pH 7.4) solution containing 0.1% Triton X-100 was supplemented with 20 μL of supernatants. The quantity of enzymes that catalyze the oxidation of 1 μM NADPH per minute is referred to as the GPx activity (U/mL). Moreover, the thiobarbituric acid reactive substance (TBAR) method, as demonstrated by Maltez et al. [26], was implemented to determine the malondialdehyde (MDA) content. At 532 nm (OD), the iMark™ Microplate Absorbance Reader (Bio-Rad) was used to quantify the MDA activity. The analysis was performed using an extinction coefficient of 156,000 M/cm.

### 2.8. Fermentation End-Products

The pH values were taken immediately upon opening the bottles each time using a pH meter (Portable pH/mV Meter-HI8424, HANNA Instruments, Singapore). Following centrifugation of the samples at 16,000× *g* for 15 min and subsequent filtering through cheesecloth, the supernatant (20 mL) was combined with 5 mL of 1 M H_2_SO_4_ and kept at −20 °C. The volatile fatty acid (VFA) and ammonia-nitrogen (NH_3_-N) content was analyzed via the following. For VFA analysis, the supernatant was subsequently filtered through a 0.45 micron syringe filter. Gas chromatography (GC) (Model HP6890, Hewlett-Packard Co., Ltd., Wilmington, DE, USA) was used for the analysis of VFA. A thirteenfold molecular sieve with a thirty-six-micron mesh size was used to pack the experimental column (Alltech Associates Inc., Deerfield, IL, USA), as described by Chuntrakort et al. [27]. For NH_3_-N analysis, the NH_3_-N content was ascertained using a LabAssay™ ammonia kit (Fujifilm Wako Pure Chemical Corp., Chuo-Ku, Osaka, Japan) and a spectrophotometer (UV/VIS, PG Instruments Ltd., Lutterworth, LEI, UK). The plate was read at an optical density of 630 nm using a microplate reader (SH-1000 Lab, Corona Electric Co., Ltd., Hitachinaka-Shi, Ibaraki-Ken, Japan).

### 2.9. Methane Production

Methane gas production was measured from total gas production at approximately 3 mL using a 10 cc glass precision syringe and stored in a 10 mL bottle. The required standard methane gas concentration was calculated using the equation C_1_V_1_ = C_2_V_2_, where C_1_ = initial concentration of standard gas (%), V_1_ = initial volume of standard gas (mL), C_2_ = desired concentration of standard gas (%), and V_2_ = desired volume of standard gas (mL). The analysis of methane production was carried out using GC equipment (Model GC-17A System, Shimadzu Co. Ltd., Nakagyo-Ku, Kyoto, Japan), with a thermal conductivity detector (injection temperature at 130 °C) and a 2 m stainless steel column packed with Shin carbon (GL science Inc., Shinjuku-Ku, Tokyo, Japan; dimensions 3 m × 3 mm, column temperature at 120 °C). The carrier gas was argon with a flow rate of 25 mL/min. Then, the standard graph of methane gas was plotted, showing the relationship between concentration and peak area obtained from GC. From this plot, the linear equation in the form of y = ax + b, where a = slope and b = y-intercept, was derived. Methane production (%) was calculated using the formula: (Peak area/18,108)/0.3, where 18,108 = the slope calculations of the standard methane graph and 0.3 = the volume of gas retained in the bottle (10 mL), following the method described by Pattra et al. [28].

### 2.10. Statistical Analysis

In accordance with SAS procedures (Software version 9.4; SAS Institute, Cary, NC, USA), the data were analyzed using the Proc Mixed method for a completely randomized design. Tukey’s test was implemented to assess the treatment’s mean. Orthogonal polynomials were employed to analyze the responses to BME addition. The treatment means were pronounced statistically significant when the *p*-values were less than 0.05 and 0.01.

## 3. Results

### 3.1. Digestive Enzyme Activity and Nutrient Degradability

The effect of BME supplementation on digestive enzyme activity and nutrient degradability are shown in Table 2. The findings indicate that BME addition increased amylase activity (linear effect; *p* < 0.01 at 12 h and *p* < 0.05 at 24 h) in the rumen when compared to the control group. However, supplementation with BME did not influence lipase activity (*p* > 0.05). Nutrient degradability, as measured by IVDMD, was significantly increased (linear effect; *p* < 0.01 at 12 h and *p* < 0.05 at 24 h) by BME supplementation, especially at 4% of the total DM substrate.

### 3.2. Antioxidant Capacity and Lipid Peroxidation

As presented in Table 3, the results revealed that BME at 4% of the total DM substrate exhibited substantially higher levels of TAOs (linear effect; *p* < 0.01 at 12 and 24 h) compared with the other treatments. The rumen antioxidant capacity of CAT was substantially increased (linear effect; *p* < 0.01) at 12 and 24 h, whereas GPx did not alter (*p* > 0.05) when treated with BME. Importantly, the treatment with diets supplemented with BME at 4% of the total DM substrate presented reduced MDA at 12 and 24 h (linear effect; *p* < 0.05) compared to other treatments.

### 3.3. Volatile Fatty Acids

The research findings found that, in comparison with the control group, BME supplementation significantly lowered acetate (linear effect; *p* < 0.01 at 12 h) and the acetate-to-propionate ratio (linear effect; *p* < 0.01 at 12 h and cubic effect; *p* < 0.01 at 24 h), while increasing propionate (linear effect; *p* < 0.01 at 12 h and cubic effect; *p* < 0.05 at 24 h) and total VFA production (linear effect; *p* < 0.05 at 12 h and quadratic effect; *p* < 0.05 at 24 h). Nevertheless, butyrate production at 12 and 24 h were similar (*p* > 0.05) among treatments (Table 4).

### 3.4. Ammonia-Nitrogen, pH, and Methane Production

No substantial differences were detected in the amounts of NH_3_-N (ranged from 19.5 to 23.2 mg/dL) and pH (ranged from 6.84 to 6.99) among the various experimental treatments (*p* > 0.05). However, there was a decrease in methane production (linear effect; *p* < 0.05 at 12 and 24 h) with the inclusion of higher levels of BME (Table 5).

## 4. Discussion

In this investigation, BME enhanced the activity of digestive exogenous enzymes, particularly amylase activity in the rumen. This may be ascribed to the PTNs abundant in BME that affect the activity of digestive enzymes from microbes. These findings agree with Sujani and Seresinhe [29], demonstrating that the rumen ecology can be altered by PTN supplements, which can influence digestive enzyme activity in ruminants. This may enhance the production of amylolytic enzymes, thereby improving digestive health and optimizing nutrient utilization. Moreover, phenolics and flavonoids can interact with and precipitate proteins, notably digestive exogenous enzymes produced by ruminal bacteria. This binding may take place at the enzyme’s active site or result in complex formations, which affect the enzymes’ potential to hydrolyze feed components. Thus, they preserve dietary protein from significant degradation by rumen microbes [30].

Enhanced IVDMD at 12 and 24 h was noted with BME supplementation, possibly attributable to BME’s richness in flavonoids and phenolic compounds, which influence ruminal microbes and microbial activity, resulting in greater degradability [31]. According to Phupaboon et al. [32], in vitro nutrient degradability can be improved by the supplementation of plant-rich PTNs (phenolics and flavonoids).

Biochemical and physiological reactions generate free radicals with an oxygen core and reactive oxygen species (ROS) as byproducts [33]. Elevated levels of ROS, resulting from either enhanced synthesis of oxidants or diminished effectiveness of the antioxidant system, can induce oxidative stress, an increasing health risk factor associated with numerous diseases in animals. There is a widespread understanding that antioxidant enzymes (CAT and GPx) and TAOs are essential for the enzymatic defense of microorganisms against oxidative stress [34], and these enzymes serve as crucial buffers against the interference and degradation of superoxide anion and hydrogen peroxide within cells [35]. MDA is generated as the end result of lipid peroxidation, and its concentration is frequently regarded as a marker of aging and oxidative damage. It may interact with biological substances and induce genotoxic and cytotoxic effects in organisms [36]. In this study, in vitro rumen antioxidant capacity of TAOs and CAT improved with BME supplementation, while MDA decreased. Accordingly, Tian et al. [37] stated that sorghum stalks possess PTNs that increase in vitro rumen antioxidant activity, specifically TAOs, GPx, and CAT. Moreover, Jia et al. [13] found that the incorporation of *Agaricus bisporus* mushroom led to decreased levels of MDA in dairy heifers, suggesting that peroxidation was significantly reduced. An increase in antioxidant enzymes was necessary for the activation of oxygen free radicals, which was accompanied by a decrease in MDA concentrations.

The generation of VFAs is the final product of the diverse metabolic process that involves the degradation and subsequent fermentation of consumed feed within the rumen [38]. Metabolic hydrogen is produced as a result of the fermentation of carbohydrates into pyruvate in the rumen. VFAs are organic hydrogen sinks that stabilize the hydrogen equilibrium, thereby facilitating fermentation [39]. The current study reported that the production of propionate and total VFAs were both enhanced by BME feeding. Phytonutrients enhance fermentation efficiency by increasing propionate production at the expense of acetate. Additionally, enhanced propionate synthesis diverts hydrogen from methanogenesis, consequently diminishing methane emissions [40]. Prachumchai et al. [41] revealed that the concentration of propionate was enriched among various levels of tropical plants abundant in PTNs by using in vitro investigation.

The enhancement of fermentation in the rumen indicates that PTNs had no effect on the rumen NH_3_-N and pH values in this experiment. Nevertheless, the NH_3_-N concentration in this study was at an acceptable level, as described by Wanapat and Pimpa [42]; they stated that NH_3_-N concentrations between 15 and 30 mg/dL were suitable for microbial protein synthesis, thereby enhancing rumen fermentation efficiency. Furthermore, consistent pH levels can promote beneficial fermentation outcomes by preserving the environment in the rumen, as demonstrated by the range of 6.5 to 7.0 [43]. The ability to enhance rumen efficiency via PTN treatment is ascribed to its capability to maintain pH levels [44].

One of the fundamental factors that contributes to inter-animal variation in enteric methane emissions is the quantity of digestible diet that an animal ingests. Enteric methane is primarily produced through hydrogenotrophic methanogenesis, which involves the reduction of carbon dioxide by hydrogen produced during the ruminal fermentation of feed [45]. Under this research, BME supplementation clearly reduced methane production. The decrease in methane may be due to the influence of PTN supplementation. Specific fermentation parameters are markedly improved when PTNs (phenolics and flavonoids) interact with methanogenesis [6]; thus, PTNs are employed for their ability to modulate methane production. One of the most significant reasons is that a decrease in ruminal methanogenesis could result in a change in rumen fermentation from acetate to propionate, a transition which is related to the preferred activation of the propionate synthesis pathway over the acetate synthesis pathway. Alter the rumen ecology by indirectly decreasing the availability of H_2_ as a substrate to compete with methanogens for accessible H_2_ [46]. Likewise, Sommai et al. [47] found that the incorporation of PTNs as a feed additive could increase propionate content, while reducing methane emissions.

The major limitation of this study is its in vitro approach, which may insufficiently reflect the intricacies of in vivo rumen dynamics and animal responses. Subsequent investigations ought to concentrate on corroborating these results in vivo to evaluate animal performance, feed efficiency, methane emissions, and possible effects on product quality.

## 5. Conclusions

The findings of the current study suggest that the addition of BME at a concentration of 4% of the total DM substrate significantly increase in vitro rumen amylase enzymes, nutrient degradability, rumen antioxidant enzymes, and lipid peroxidation. BME supplementation could increase propionate and total VFA production, while reducing methane production. Therefore, BME, an alternative dietary mushroom-based PTN, has the ability to efficiently enhance rumen enzymes, antioxidant capacity, and fermentation end-products.

## Figures and Tables

**Table 1:** Ingredients and nutritional composition of diets.

Items	Concentrate	Rice Straw	Bolete Mushroom Extract
**Ingredients (% as fed)**			
Cassava chip	59.2		
Soybean meal	15.0		
Palm kernel meal	13.0		
Rice bran meal	9.0		
Urea	1.3		
Premixed minerals ^1^	1.0		
Salt	1.0		
Sulphur	0.5		
**Chemical compositions**			
Dry matter (DM, %)	92.6	94.4	99.4
	--------------------------------% Dry matter--------------------------------		
Organic matter (OM)	88.8	82.8	87.8
Crude protein (CP)	14.1	2.7	2.1
Neutral-detergent fiber (NDF)	15.9	78.1	0.23
Acid-detergent fiber (ADF)	8.6	53.9	0.08
**Phytonutrient compounds**			
TPC (mg GAE/g DM)	-	-	3.2
TFC (mg QUE/g DM)	-	-	0.2

TPC, total phenolic compounds; TFC, total flavonoid compounds; GAE, gallic acid equivalent; QUE, quercetin. ^1^ Premixed minerals (contents per kg): vitamin A 10,000,000 IU; vitamin D 1,600,000 IU; vitamin E 70,000 IU; Fe 50 g; Mn 40 g; Zn 40 g; Cu 10 g; I 0.5 g; Se 0.1 g; Co 0.1 g.

**Table 2:** Effect of bolete mushroom extract on digestive enzyme activity and nutrient degradability.

Treatments	BME	Rumen Enzymes (U/mL)						IVDMD (% DM)	
		Amylase		Lipase		Protease			
		12 h	24 h	12 h	24 h	12 h	24 h	12 h	24 h
1	0	6.2 ^c^	6.3 ^c^	31.0	30.9	26.5	26.5	58.2 ^e^	67.4 ^c^
2	1	6.3 ^b^	6.4 ^b^	30.9	31.1	26.8	26.8	60.2 ^de^	67.6 ^c^
3	2	6.4 ^a^	6.5 ^a^	31.2	31.2	26.6	26.8	62.5 ^bc^	67.5 ^c^
4	3	6.4 ^a^	6.5 ^a^	30.8	31.0	26.8	26.8	63.3 ^b^	70.4 ^b^
5	4	6.4 ^a^	6.5 ^a^	30.9	31.1	26.8	26.8	66.2 ^a^	73.4 ^a^
6	5	6.2 ^c^	6.3 ^c^	31.0	31.0	26.7	26.7	61.0 ^cd^	70.7 ^b^
SEM		0.01	0.02	0.04	0.03	0.02	0.04	0.17	0.18
**Orthogonal polynomials**									
Linear		<0.01	0.03	0.91	0.52	0.16	0.14	<0.01	0.01
Quadratic		0.28	0.77	0.35	0.22	0.64	0.39	0.37	0.07
Cubic		0.54	0.96	0.15	0.53	0.08	0.55	0.52	0.27

BME, bolete mushroom extract (% of total DM substrate); IVDMD, in vitro dry matter degradability; SEM, standard error of mean. ^a–e^ Means within the same column with different letters are significantly different at *p* < 0.05.

**Table 3:** Effect of bolete mushroom extract on rumen antioxidant capacity and lipid peroxidation.

Treatments	BME	Rumen Antioxidant Enzymes				TAOs		MDA	
		CAT		GPx					
		12 h	24 h	12 h	24 h	12 h	24 h	12 h	24 h
1	0	3.9 ^c^	5.7 ^c^	12.3	13.8	83.9 ^c^	83.5 ^d^	18.9 ^a^	18.6 ^a^
2	1	8.0 ^b^	11.7 ^b^	13.4	14.6	85.3 ^ab^	85.6 ^b^	17.3 ^ab^	17.2 ^ab^
3	2	13.8 ^a^	14.7 ^ab^	13.9	14.4	85.2 ^b^	85.7 ^b^	16.6 ^abc^	17.6 ^ab^
4	3	13.9 ^a^	14.9 ^ab^	13.0	14.3	85.9 ^ab^	86.3 ^a^	16.6 ^abc^	17.4 ^ab^
5	4	15.7 ^a^	18.0 ^a^	13.7	14.3	86.3 ^a^	86.5 ^a^	14.5 ^c^	16.7 ^b^
6	5	15.9 ^a^	15.5 ^ab^	13.8	14.3	85.7 ^ab^	84.6 ^c^	15.7 ^bc^	17.4 ^ab^
SEM		0.35	0.46	0.15	0.08	0.07	0.04	0.20	0.10
**Orthogonal polynomials**									
Linear		<0.01	<0.01	0.31	0.29	<0.01	<0.01	0.04	0.03
Quadratic		0.16	0.12	0.11	0.16	0.16	<0.01	0.30	0.15
Cubic		0.23	0.97	0.77	0.38	0.10	0.91	0.96	0.18

BME, bolete mushroom extract (% of total DM substrate); CAT, catalase (U/mL); GPx, glutathione peroxidase (U/mL); TAOs, total antioxidants (% inhibition); MDA, malondialdehyde (nmol/mL); SEM, standard error of mean. ^a–d^ Means within the same column with different letters are significantly different at *p* < 0.05.

**Table 4:** Effect of bolete mushroom extract on volatile fatty acid production.

Treatments	BME	Volatile Fatty Acids (mol/100 mL)						C_2_:C_3_		Total VFAs (mmol/L)	
		C_2_		C_3_		C_4_					
		12 h	24 h	12 h	24 h	12 h	24 h	12 h	24 h	12 h	24 h
1	0	75.2 ^a^	73.9	23.0 ^b^	24.4 ^d^	1.8	1.7	3.3 ^a^	3.0 ^a^	52.6 ^b^	68.7 ^d^
2	1	73.7 ^ab^	72.6	24.7 ^ab^	25.8 ^c^	1.6	1.6	3.0 ^b^	2.8 ^c^	55.2 ^ab^	73.4 ^c^
3	2	73.6 ^ab^	73.3	24.8 ^a^	25.1 ^d^	1.6	1.6	3.0 ^b^	2.9 ^b^	57.1 ^a^	76.8 ^b^
4	3	72.7 ^b^	73.3	25.9 ^a^	25.0 ^d^	1.4	1.7	2.8 ^c^	2.9 ^b^	57.8 ^a^	77.1 ^ab^
5	4	72.4 ^b^	69.1	26.3 ^a^	29.1 ^a^	1.2	1.8	2.8 ^c^	2.4 ^e^	58.5 ^a^	79.4 ^a^
6	5	73.6 ^ab^	71.9	24.9 ^a^	26.5 ^b^	1.5	1.6	3.0 ^b^	2.7 ^d^	56.2 ^ab^	76.5 ^b^
SEM		0.13	0.14	0.13	0.04	0.04	0.03	0.02	0.01	0.33	0.19
**Orthogonal polynomials**											
Linear		<0.01	0.60	<0.01	0.31	0.06	0.74	<0.01	0.60	0.01	<0.01
Quadratic		0.50	0.20	0.55	<0.01	0.72	0.71	0.13	<0.01	0.45	0.01
Cubic		0.35	0.27	0.32	0.02	0.63	0.86	0.28	<0.01	0.90	0.55

BME, bolete mushroom extract (% of total DM substrate); VFAs, volatile fatty acids; C_2_, acetate; C_3_, propionate; C_4_, butyrate; C_2_:C_3_, acetate-to-propionate ratio; SEM, standard error of mean. ^a–e^ Means within the same column with different letters are significantly different at *p* < 0.05.

**Table 5:** Effect of bolete mushroom extract on pH, ammonia-nitrogen, and methane production.

Treatments	BME	pH		Ammonia-Nitrogen (mg/dL)		Methane Production (%)	
		12 h	24 h	12 h	24 h	12 h	24 h
1	0	6.9	6.9	19.5	21.7	12.3 ^a^	16.4 ^a^
2	1	6.9	6.8	20.7	22.3	11.5 ^ab^	15.7 ^ab^
3	2	6.9	6.8	20.8	22.1	11.5 ^ab^	15.5 ^ab^
4	3	6.9	6.8	20.9	22.5	10.8 ^bcd^	14.3 ^bc^
5	4	6.9	6.9	20.6	23.0	9.6 ^d^	13.3 ^c^
6	5	6.9	6.9	20.3	23.2	10.3 ^cd^	14.2 ^bc^
SEM		0.01	0.01	0.17	0.14	0.09	0.12
**Orthogonal polynomials**							
Linear		0.87	0.21	0.14	0.42	0.02	0.01
Quadratic		0.31	0.75	0.39	0.88	1.00	0.64
Cubic		0.29	0.58	0.64	0.63	0.29	0.51

BME, bolete mushroom extract (% of total DM substrate); SEM, standard error of mean. ^a–d^ Means within the same column with different letters are significantly different at *p* < 0.05.

## Data Availability

The original contributions presented in this study are included in the article. Further inquiries can be directed to the corresponding author.
